# Policies on sexual expression in forensic psychiatric settings in different European countries

**DOI:** 10.1186/s13033-016-0037-y

**Published:** 2016-02-03

**Authors:** Rajveer Tiwana, Stephanie McDonald, Birgit Völlm

**Affiliations:** School of Psychology, University of Nottingham, Nottingham, NG7 2RD UK; School of Psychology, University of Nottingham, Nottingham, NG9 2RD UK; Section Forensic Mental Health, Division of Psychiatry and Applied Psychology, Institute of Mental Health, University of Nottingham, Triumph Rd, Nottingham, NG7 2TU UK

**Keywords:** Sexual expression, Forensic psychiatric hospitals, European policies, Long-stay patients, Mental health, Patient relationships, Human rights

## Abstract

**Background:**

Sexual expression by forensic psychiatric patients is poorly researched.

**Methods:**

Forensic experts representing 14 European countries were interviewed to explore the diverse ways in which sexual expression within forensic settings is handled.

**Results:**

No country had a national policy, although many had local policies or shared practices. Progressive approaches to patient sexuality were evident in nine of the countries sampled. The UK appeared the most prohibiting and excluding, its protocols apparently based on risk aversion and lack of emphasis or consideration of patients’ sexual needs.

**Conclusions:**

Uniform national policy supporting patients’ sexual expression would provide significant improvements.

**Electronic supplementary material:**

The online version of this article (doi:10.1186/s13033-016-0037-y) contains supplementary material, which is available to authorised users.

## Background

A significant proportion of mentally disordered offenders require long-term care within a forensic-psychiatric hospital [1, 2]. There is currently interest in defining more clearly the characteristics and needs of this population [3], but to date the management of sexual expression by (long-stay) forensic inpatients has received little attention.

It has been suggested that sexuality involves *“the totality of being a person”* [4], and that the right to sexuality is a fundamental aspect of the human condition [5]. Perlin and Lynch [6] suggest that individuals with mental health disabilities have the same needs for intimate relationships as everyone else, and that denying such patients their sexuality undermines their rights and threatens their psychological well-being. Furthermore, there is evidence that sexual and relationship well-being can enhance the recovery process, can act as a positive behavioural motivation, and can help resolve historical emotional and psychological problems [1].

There can be advantages in helping to maintain an established relationship with a long-term partner. In a prison setting, for example, the Howard League for Penal Reform in the UK recently concluded that maintaining contact with partners and families on the outside can reduce reoffending and help with prisoners’ rehabilitation [7]. Conversely, prohibiting a patient who is compulsorily detained in a forensic unit from engaging in sexual activity with a long-term partner from outside the institution is likely to put a severe strain on their relationship, increasing the chance of relationship breakdown, psychological disturbance and less motivation to leave hospital, all of which inevitably hinders the recovery process. There is also an argument for allowing sexual relationships between the person detained and his or her peers, since the experience of a restrictive policy may affect how a patient understands and anticipates sexuality and relationships. As well as undermining existing relationships, this can lead to difficulties initiating new ones, and disrupt the return to a normal sex life upon release, which in turn increases potential for mental and criminal relapse [1, 4].

Whilst restrictive policies on sexual expression affect forensic and non-forensic patients alike, these restrictions impact disproportionately on those in forensic settings due to their long, sometimes life-long detention. In addition, deprivation of privacy and restrictions on sexual relationships may be key factors in the relationship failures that are often seen in forensic psychiatric patients [5]. Imposing a restriction on a person’s sexual relationships may impinge on his or her rights under Article 8 of the European Convention on Human Rights (ECHR); this upholds the right to privacy, personal dignity, autonomy and social interaction even when involuntarily detained. Bartlett and colleagues [5] have observed that curtailment of sexual activity or the right to form relationships with others can only be justified if it is ‘in accordance with the law’ and ‘necessary in a democratic society’ in the interests of stated factors. These factors might include the prevention of disorder or crime, health, and the protection of rights and freedoms of others, with the possibility that such restrictive policies could be defensible under article 8(2) of the ECHR for some institutions within the high secure sector. It would not be easy to argue for a policy of blanket prohibition, however. An individualised patient-centred approach would effectively be required as a matter of law, allowing a patient to challenge decisions made restricting his or her rights to engage in sexual and emotional relationships [5].

Information is, however, currently lacking on how sexual expression by forensic psychiatric patients is managed in different European countries, and we were unable to identify any comparative study. Some considerable variation in policies and procedures might reasonably be anticipated, given that the placement and treatment of mentally disordered offenders in the European Union Member States is characterised by a considerable variety of concepts and practices [8], attributable in part to differences in professional guidelines [9], legal frameworks, policies, and resources [2, 10–13].

In the UK, for example, there is no national policy on sexual expression amongst forensic patients [4]. Management decisions are left to individual hospitals which tend to adopt a default position of sexual exclusion. Various explanations for this have been suggested, including an emphasis on the ethical and litigious dangers involved, concerns regarding family, public and media disapproval, lack of private space, inadequate staffing, insufficient training on patient sexuality and viewing forensic patients as undeserving of sexual freedom as is the case with offenders more generally [1, 4, 6, 8, 14–16]. As a result, inpatient sexual activity is effectively prohibited in the UK. Any management issues that arise are left to the judgment of mental health staff who may respond arbitrarily and inconsistently to patients driven into dangerous clandestine sexual activity with the associated risks of exploitation, sexually transmitted disease and unexpected pregnancy [4, 5, 8, 17].

Not all approaches to sexual expression focus on exclusion, however. A discourse amongst treatment providers and policymakers is developing that recognises the need for more progressive policies on sexuality that go beyond perceiving sexual expression in terms of risk control [5, 14]. There is also interest in the therapeutic recovery potential that may result from valuing intimacy and affection [12, 16, 18].

This study seeks to explore the various ways in which sexual expression by forensic psychiatric patients is currently managed across Europe.

## Methods

### Objective and design

The aim of this study was to explore the diversity of ways in which sexual expression by forensic psychiatric patients is handled in different European countries; we sought to explore contrasts between the various approaches rather than attempting to identify the general situation across Europe. This was achieved by asking experts in forensic psychiatry from fourteen European Union-Member States to summarise the current situation in their country by responding to a set of structured questions (via an online questionnaire using Surveymonkey) and, for a subset of participants, to a set of semi-structured questions (via telephone interview).

### Participants

Fourteen participants, each representing a different country, were drawn from a European network of experts in forensic psychiatry (COST-action; http://www.lfpc-cost.eu/). This network comprises leading clinicians and researchers with expertise in forensic psychiatry and a particular interest in long-term forensic psychiatric care. Each expert participant had extensive experience working in secure forensic psychiatric hospitals and was familiar with the policies or guidelines relating to patient relationships and sexual expression in his or her country, where these existed. The countries represented were Austria, Belgium, Denmark, Finland, FYR Macedonia, Germany, Italy, Latvia, Lithuania, the Netherlands, Scotland, Spain, Switzerland and the UK.

Representatives from four of the countries (Germany, Netherlands, Switzerland and the UK) agreed to participate in an additional telephone interview designed to elicit greater detail. These countries were chosen because of their diversity: the UK and the Netherlands representing the more extreme ends of restrictiveness/permissiveness in forensic psychiatric care, with the other two representing more of a middle ground. All these countries are also known for their long history of forensic-psychiatric care provision. An additional expert from the UK was invited to participate in the telephone interviews to allow both the medium and the high secure forensic environments that exist in that country to be represented.

### Procedure

Each participant gave their informed consent and responded electronically to a set of structured questions using an online survey tool (http://www.surveymonkey.co.uk). The questions (see Additional file 1) covered any policies relating to patients having sexual intercourse or intimate contact with other patients or with partners outside the institution, the resources available to patients (such as access to conjugal visiting suites and contraception), any relevant staff training and patient awareness and involvement in the development of the policy. Participants were encouraged to make additional comments in their response to each of the questions if they so wished. The telephone interviews were conducted by RT and comprised a set of semi-structured questions (see Additional file 2) which were individualised to each participant based on the previous email responses. Interviews lasted between 20 and 60 min and were audio recorded (with the participant’s permission) and subsequently transcribed verbatim.

### Analysis

Online responses were summarised narratively and tabulated. Transcripts from the semi-structured interviews were subjected to thematic analysis by two independent raters to identify common emergent themes and the positions taken by each country. The thematic analysis followed the six phases recommended by Braun and Clarke [19]: familiarisation with the data; generating initial codes; searching for themes; reviewing themes; defining and naming themes; and reporting the analysis. Inter-rater reliability was estimated from three interviews, based on the ratio of the number of themes agreed upon compared to the number disagreed upon.

## Results

### Online questionnaire

Responses from experts from the ten countries are summarised narratively in Table 1. Four participants (representing Austria, Italy, Lithuania and Scotland) found upon closer inspection of the questions that their country had little or no developed policy or shared practise, so felt they were unable to contribute meaningfully to the study. These countries are not included in Table 1.

**Table 1:** Summary of responses to online questionnaire

Country	Application of policy rules	Direction	Other forms of expression allowed	Patient/visitor interaction	Resources available to patients	Staff training	Patient awareness and involvement
Belgium	Hetero- & homosexual relationships treated the same Same policies regardless of case history	Sexual intercourse prohibited inside institution Romantic/non-sexual relationships between patients prohibited Patients can marry	Holding hands Access to sexually explicit DVDs, magazines & novels	Patients not allowed into each other’s bedrooms Visitors allowed unsupervised access to patients’ bedrooms ‘Blind eye’ turned to potential sexual activity with spouse No conjugal visiting suite	Sexual education Condoms/contraception	Provided	No written policy; patients informed verbally Patients have opportunity to complain about policy Patient perspective not considered
Denmark	Hetero- & homosexual relationships treated the same Decided on case-by-case basis	Sexual relationships with existing long-term partner permitted^a^ Sexual and non-sexual relationships between patients prohibited Patients can marry	Access to sexually explicit DVDs, magazines & novels	Patients not allowed in each other’s bedrooms Visitors not allowed in patients’ bedrooms Conjugal visiting suite available	Condoms/contraception	No specific training	No written policy; patients informed verbally Patients have opportunity to complain about policy Patient perspective not considered
Finland	Hetero- & homosexual relationships treated the same Same policies regardless of case history	Sexual relationships permitted^a^ between patients or between patient and outside partner, including prostitutes Non-sexual relationships supported and seen as positive and prosocial Patients can marry	Kissing Hugging Caressing Massage Holding hands Access to sexually explicit DVD’s, adult magazines, erotic novels & pornographic websites	Patients not allowed into each other’s bedrooms Visitors not allowed into patient bedrooms No conjugal suite Visits take place in visiting area and can be unsupervised	Relationship counselling Sexual education Condoms/contraception	No specific training	No written policy; patients informed verbally Patients have opportunity to complain about policy Patient perspective not considered
FYR Macedonia	Homosexual relationships not taken into consideration Decided on case-by-case basis	Sexual relationships permitted^a^ between patient and existing long-term partner Sexual relationships between patients prohibited Non-sexual relationships allowed Patients cannot marry	Kissing Hugging Caressing Massage Holding hands Access to adult magazines	Patients allowed supervised access to each other’s bedrooms Visitors allowed unsupervised access to patient bedrooms^a^	Relationship counselling^a^ Condoms/contraception	No specific training	Not available for patients to see Patient perspective not considered
Germany	Hetero- & homosexual relationships treated the same Decided on case-by-case basis	Sexual relationships permitted^a^ between patient and existing long-term partner, and new partner outside the institution, including prostitutes Sexual relationships between patients prohibited Non-sexual relationships allowed^a^ Patients can marry	Kissing Hugging Caressing Massage Holding hands Access to adult magazines & erotic novels	Patients allowed unsupervised access to each other’s rooms^a^ Visitors allowed unsupervised access to patient bedrooms^a^ Conjugal visiting suite available	Relationship counselling Sexual education Condoms/contraception	Provided	Policy readily available for patients to see Patient perspective not considered
Latvia	Hetero- & homosexual relationships treated the same Same policies regardless of case history	Sexual intercourse prohibited within unit Sexual relationships permitted with partners from outside the institution Non-sexual relationships allowed^a^ Patients can marry	No form of sexual expression allowed	Patients not allowed into each other’s bedrooms Visitors not allowed into patient bedrooms Conjugal visiting suite available in some facilities	None	Not known	Policy available for patients to see Patient perspective not considered
Netherlands	Hetero- & homosexual relationships treated the same Decided on case-by-case basis	Sexual relationships permitted^a^ between patient and existing long-term partner, and new partner outside the institution, including prostitutes Non-sexual relationships allowed^a^ Patients can marry^a^	Kissing Hugging Caressing Massage Holding hands Access to sexually explicit DVD’s, adult magazines, erotic novels allowed^a^	Patients allowed unsupervised access to each other’s bedrooms Visitors allowed unsupervised access to patient bedrooms^a^ Conjugal visiting suite available	Relationship counselling Sexual education Condoms/contraception	General training provided	Policy available for patients to see Patient perspective not considered
Spain	Not known	Sexual relationships permitted^a^ between patient and existing long-term partner Non-sexual relationships allowed^a^ Patients can marry	Kissing Hugging Caressing Massage Holding hands Access to erotic novels	Patients not allowed into each other’s bedrooms Visitors not allowed into patient bedrooms Conjugal visiting suite available	Sexual education	No specific training	Not readily available for patients to see Patient perspective not considered
Switzerland	Hetero- & homosexual relationships treated the same Same policies regardless of case history	Sexual relationships discouraged amongst patients Sexual relationships permitted between patient and existing long-term partner, and new partner Non-sexual relationships allowed^a^ Patients can marry	Kissing Hugging Caressing Massage Holding hands No access to sexually explicit DVD’s, adult magazines, erotic novels or pornographic websites	Patients not allowed into each other’s bedrooms Visitors not allowed into patient bedrooms Sexual activity with partners allowed when patient is on leave	Relationship counselling Sexual education Condoms/contraception	Provided	Not readily available for patients to see Patient perspective considered in drafting of shared practice
United Kingdom	Hetero- & homosexual relationships treated the same Officially, decided on case-by-case basis but in practice policy implemented uniformly	Sexual relationships between patients and between patient and partner outside the institution prohibited Patients can marry	Minor forms of physical contact but no intimate or extended contact Access to erotic novels allowed^a^	Patients not allowed into each other’s bedrooms Visitors not allowed into patient bedrooms Conjugal visiting suite not available	Relationship counselling Sexual education^a^	No specific training	Abbreviated version available for patients to see Patient perspective not considered

^a^depending on circumstance

A striking finding is that none of the fourteen countries has a national policy. Five countries had local policies for specific medium or high secure settings: Belgium, Denmark, Finland, Netherlands and the UK. Representatives from five countries reported shared practice: Latvia (nationally), Spain (nationally), Switzerland (nationally), Netherlands (high & medium secure hospitals) and FYR Macedonia (high secure units in the South West). The 10 countries listed in Table 1 thus had hospital specific policies, a local regional policy, or a shared practice adopted across different units. Respondents were therefore asked to respond to questions in relation to their own hospital’s policy or wider shared practice where available.

The UK alone stood out as providing no opportunity for patients to have sexual relationships with a partner either inside or outside the institution, whereas the other nine countries permit sexual relationships between patients or between patients and an outside partner, or both. Access to conjugal suites is available in Denmark, Germany, Netherlands and Spain, and in Latvia depending on the capacity of the facility. Finland provides a visiting area for sexual activity for qualifying patients. FYR Macedonia and Belgium allow visitors unsupervised access to patients’ bedrooms which may include sexual activity, whereas Switzerland does not allow sexual intercourse within the hospital but appears to be open to this happening if a patient is on leave.

There was a tendency for sexual relationships between patients to be prohibited or more strongly discouraged than was the case for a sexual relationship with a partner outside the institution. In our sample, Finland is the only country allowing patients to form sexual relationships with each other, in certain circumstances. Germany and Netherlands do, however, allow patients to spend time in other’s rooms unsupervised where opportunities for sexual activity could arise.

Each of the countries in Table 1 granted patients the right to get married, with the exception of FYR Macedonia. Interestingly, forensic patients in the UK have the legal right to marry but not to consummate that marriage. Each country applies the same policy to both heterosexual relationships and homosexual relationships, with the exception of FYR Macedonia where homosexual relationships are not acknowledged. With the exception of Latvia, the physical expression of affection (such as hugging and holding hands) is allowed in each of the countries to varying degree, as is masturbation as long as this is in private. Latvia does not allow any form of sexual or affectionate expression, possibly because 6–7 patients often share one bedroom and therefore have no privacy.

With the exception of Spain, all countries listed in Table 1 give patients access to condoms and contraception (although the UK did not specify on this). Belgium and Switzerland are the only countries providing staff with specific training on patient sexual expression. None of the countries, with the exception of Switzerland, had considered the patients’ perspective when devising their policy or shared practice on sexual expression.

### Telephone interviews

Inter-rater reliability was calculated as 70.6 %, which exceeds the minimum figure suggested by Marques and McCall [20]. A total of eight themes were generated from the coded data (see Table 2).

**Table 2:** *Emergent themes from the telephone interviews*

Themes	Definition	Sub themes
1. General views on sexual expression^b, c, d, e^	Stance on condoning the expression of sexuality and intimacy and patients having relationships in secure forensic-psychiatric care	Positive^b, c^ Negative^d, e^
2. Screening procedures^a, b, c, d, e^	Procedures put in place to screen for any risks or vulnerabilities related to sexual activities. These could include sexual contact with another patient, a partner outside the institution (this can be a long-standing partner, a new partner, a prostitute)	Relationship with partner outside the institution^a, b, c, e^ Vulnerability of patient^a, b, c, e^ Relationship between patients^a, b, c^
3. Safe sex^a, b, c, d^	Safe sex put in place when considering patients being sexually active	Distributing condoms^a, b, c, d^ Access to contraception^a, b^ Sexually transmitted diseases^a, c^
4. Private space^a, b, c, d, e^	Patients having privacy in a designated area to carry out sexual activity	Conjugal suites^a, b, c^ Bedrooms^b, c^ On leave^a, b, e^
5. Public opinion^a, b, c, d^	Perception of public opinion on patients having sexual relationships and expressing their sexuality	Restrictive/punitive^a, b, c, d^
6. Patients’ responses^a, b, c, d, e^	Response of service users to policies on sexual expression	Few complaints^a, b, e^ Seen as restrictive^a, c, d^
7. Policy implementation^a, b, c, d, e^	Views of staff on implementation of policy on sexual expression	Considerable difficulty^a^ Occasional difficulty^c, d^ Issue not raised^b, e^
8. Future plans^a, c, d, e^	Future plans and ideas that could help in the development of policies on sexual expression in forensic-psychiatric care	Maintaining long-term relationships^d^ Male homosexual relationships^e^ Learning from others^a, d, e^ National discussions^a, c^

^a^Switzerland

^b^Germany

^c^Netherlands

^d^UK, high secure

^e^UK, medium secure

#### Theme 1: general views on sexual expression

The experts from Germany and the Netherlands made specific reference to patients as sexual and emotional beings who required relevant expression of their sexuality: *“Sexual expression is an important part of human nature”* (Netherlands) and *“It’s part of the patient’s life”* (Germany). They interpreted such expression as including non-sexual intimacy: *“walking hand*-*in*-*hand…cuddling somebody…”* (Netherlands). For forensic patients, sexual expression appeared to be respected as a human right to be restrained only *“for security reasons”* (Germany).

The expert from the Netherlands observed that sexual expression can reduce risk and be part of the therapeutic process: *“Too much repression…can even lead to exaggerated frustration and elevation of risk levels”* (Netherlands). In contrast, the UK participants viewed sexual expression as potentially problematic: *“I have concerns about it…”* (UK, medium secure) and as an unnecessary disturbance to patient management and recovery: *“…it could be interfering with the therapy or treatment or moving on…”* (UK, high secure). There was recognition of the significance of long-standing relationships: *“…maintaining long*-*term relationships is really important…”* (UK, medium secure), but also a tendency to consider such relationships as a treatment problem rather than a potential asset: *“They’ve got a wife or husband at home which is really difficult when their length of stay here could be five years”* (UK, high secure).

#### Theme 2: screening procedures

A strict screening procedure operates in all countries if a patient wants to have sexual intercourse with their partner from outside the institution. Such screening also ensures patient safeguarding: “*Both risk from the patient to others and risk of vulnerability of the patient as well…” (*UK, medium secure). In the Netherlands and Germany, the outside partner can also include a prostitute, but this too requires assessment and screening. In Switzerland, the Netherlands and Germany any relationship between patients is also screened to ensure it is safe psychologically and physically, and where risks and dangers are identified to ensure these are dealt with. In Germany, for example, if: *“…we know of a patient to have sex diseases, hepatitis C…we would say you cannot put other patients at risk.”*

In the Netherlands, a patient’s request to get married is also assessed independently to ensure neither party is being coerced: *“…assessed by a psychiatrist outside…to see if the question is out of free will”* (Netherlands).

#### Theme 3: safe sex

The countries that appear most progressive in terms of sexual expression policy, such as Germany and Netherlands, were also those most proactive in promoting safe sex procedures. In the UK and Switzerland, where sexual intercourse is forbidden, patients may be reluctant to ask for condoms/contraception and thus are in more danger of contracting STDs: *“They usually don’t ask even because they know it’s forbidden…”* (Switzerland). The issue of homosexuality is a significant factor: *“…should we issue condoms to male patients if we feel they’ve got a relationship with somebody with the same gender? But we decided that that just opened too much of a floodgate really…”* (UK, high secure). Health risks such as these appear to be addressed more proactively in the Netherlands: *“…the more pressure we put on it, the more it gets secret; then there will be more difficulties and dangers of illness.”*

The mechanics of the process differs between countries. In the Netherlands, nursing staff distribute condoms, whereas in Germany: *“…a relative or people outside send it to them…”.* For females in Germany and Switzerland, contraception usually takes the form of an injection carried out in a formal, clinical setting.

#### Theme 4: private space

Given that most bedrooms in forensic care settings are designed for single occupancy, one determinant of whether a patient gets any ‘private time’ with their partner is whether the units have commissioned suitable separate accommodation. In Germany, patients can spend private intimate time with other patients in their bedrooms when there is no private designated room. In contrast, the view in the UK is that the hospital is a public place where sexual activity is inappropriate: *“…it’s a public space so that’s why anything of intimacy would be discouraged and not acceptable”* (UK, high secure). Consequently, sex is only considered possible in the UK when patients are granted unescorted leave: “…*if they start to have their sexual relationship within their unescorted leave, that’s absolutely fine”* (UK, medium secure).

#### Theme 5: public opinion

Responses suggested that the general public’s view on patients’ sexuality tends to be restrictive and punitive in all the countries sampled. Often, perception of patients’ quality of life appears overestimated and a common view is that patients should have their freedom curtailed whilst in detention: *“…public opinion is that they have a very good life in prisons and in hospitals which is not true…so now you even allow them to have sex?”* (Switzerland). This is reinforced by notions of forensic psychiatric patients as being either sexually corrupt, or not fully credited as sexual beings: *“…they should not have sexual feelings as psychiatric patients”* (Netherlands).

#### Theme 6: patients’ responses

When interviewees were asked about how patients responded to policy on sexual expression, their replies suggested that most patients tend generally not to complain: *“… for about 80* *% it’s no problem”* (Switzerland). This may vary with the nature of the policy in operation, however. For example, Germany’s liberal policy appears to have occasioned few complaints: *“I’m not aware about one single complaint in twenty eight years since I’m a director here”* (Germany). In contrast, the UK’s rigid guidelines regarding the way people dress or act, even under supervision, are sometimes contested: *“… his girlfriend came to visit and they were a bit too intimate in front of the staff … and they were discouraged from this intimacy and she was dressed inappropriately as well and we put a stop to that, and the patient challenged that and said it’s my girlfriend”* (UK, high secure). Individual circumstances seem also to be important: one representative noted occasional complaints that the rules were too strict, but that these were *“mainly from patients who do not have a partner; patients who have a partner sometimes want more time together but are, on the whole, content”* (Netherlands).

#### Theme 7: policy implementation

A number of comments were made on the practicality of a policy, particularly from the viewpoint of staff who were charged with implementing it. Some comments suggested that implementation presented a considerable challenge: “*… [it is] very difficult for them, especially during the night to be sure that the patients don’t go to each other rooms”* (Switzerland). Others seemed to suggest that policy implementation was a challenge only occasionally. One representative reported: “*Staff sometimes find it hard because they find it hard to discuss sex as a topic, particularly homosexual relations,but this reluctance can very well be explained by their personal feelings concerning homosexuality*” (Netherlands). Another stressed the importance of discerning the nature of *“the actual relationship between patient and patient”* when attempting to implement any policy, and differentiating, for example, *“family hugs”* from a relationship that is abusive or volatile (UK high secure). In contrast, representatives from Germany and UK, medium secure, indicated they were not aware of implementation being a particular issue for staff.

#### Theme 8: future practice

Experts from Switzerland and the UK indicated that it was useful to compare their own policies on sexual expression with those of other countries with more established practices, and that they might learn from and consider adapting such measures to their own institutions. In the UK, for example, the possibility of allowing access to explicit magazines, maintaining long-term relationships and facilitating conjugal visits was not ruled out: *“I was unaware of the extent of the conjugal visits in Europe and it might be something we should debate more openly”* (UK, medium secure). There was also acknowledgement of the need to more readily acknowledge homosexual relationships amongst male patients to ensure equal treatment and to prevent STDs: *“We have men in long*-*term secure care but we don’t seem to be picking up homosexual relationships between men. Now that…needs more work on”* (UK, medium secure).

The experts from Switzerland and the Netherlands advocated a broader campaign to raise sexual consciousness in society. *“…we could use a more liberal viewpoint by politicians to be expressed in the public debate about the fact that sex is not wrong, bad or anything like that per se” (Netherlands).*

## Discussion

The purpose of this study was to explore the various ways in which sexual expression by forensic psychiatric patients is currently managed across Europe. This was achieved by questioning experts in forensic psychiatry from 14 European Union-Member States using an online questionnaire and telephone interviews. The material gathered from the forensic experts who participated in this study clearly indicates that the issue of sexual expression by forensic psychiatric patients is handled very differently between European countries. The study targeted Austria, Belgium, Denmark, Finland, FYR Macedonia, Germany, Italy, Latvia, Lithuania, the Netherlands, Scotland, Spain, Switzerland and the UK. None of these countries had a national policy on patient sexual expression, although many had unwritten shared practices or local policies to which they adhered.

The expert participants provided detailed information on policies and practices operating in ten of the targeted countries. From these, the UK stands out as being the most restrictive in comparison to the other nine who appeared to be more progressive and accommodating regarding the sexual needs of their forensic patients. A particular example of this restrictive approach is the situation in the UK where forensic patients have the legal right to marry but not to consummate that marriage, which seems to suggest that this policy merely satisfies a legal requirement.

The nine countries which allow some kind of sexual expression are, as expected, fully aware of the need to manage risk and protect vulnerable patients but, in contrast to the UK, appear to balance this with a more positive acknowledgement of patients’ rights to sexuality and relationships, especially if such a permissive approach aids the therapeutic process. The information gathered in this study suggests that these countries have not experienced significant problems resulting from this more lenient approach—for example, the representative from Germany was unable to recall any complaint arising from their policy during 28 years of being the medical director of one of the largest forensic hospitals in the country. The less restrictive policies and practices in countries such as Netherlands and Germany which incorporate patients’ sexual expression into their individual treatment plans may also be beneficial: two separate studies [18, 21] have found that intimacy and affection can have a positive impact on forensic patients’ rehabilitation and recovery.

Material gathered in the interviews suggested a wide variation in the perception of the level of difficulty faced by staff when attempting to implement a policy. This variability may itself reflect the leniency of the policy, or even the level of support they experience. One of the difficulties identified was that for some staff their personal views and feelings conflicted with the aims of the policy. A similar finding emerged in a study of nurses’ attitudes towards sexual relationships between patients within secure psychiatric hospitals in England where practices and attitudes were found to be dominated by personal values [22].

In terms of patients’ responses to local policy on sexual expression, it is interesting to note that interviewees’ comments tended to focus on the presence or absence of complaints from service users. Whilst this provides some information on their response to a policy, focusing solely on complaints does not necessarily lead to a comprehensive understanding of patients’ views on the topic. In this respect, it is important to point out that none of the countries in our sample, with the exception of Switzerland, considered the patients’ perspective when devising local policy or shared practice.

The comments from interviewees suggested that most patients tend generally not to complain about the local policy on sexual expression. This may indicate a general satisfaction, but is perhaps more likely to arise because patients within secure care tend to have limited ambition and rarely consider the possibility of challenging such a policy. For example, one interviewee observed:”*Occasionally there have been challenges, but I think patients are generally aware of what the boundaries are within high secure with what they can’t do and what’s acceptable*” (UK, high secure).

In terms of the general public’s view on patients’ sexuality, responses suggested that this tends to be restrictive and punitive across the countries sampled, and this was to some extent paralleled in the UK’s approach to sexuality in the forensic-psychiatric setting which can be viewed essentially one of restraint, exclusion and, possibly, taboo. Perceptions appear to lean heavily towards the punitive aspect of patients’ detention and the management of the patient’s sexuality is seen as problematic. In contrast, the experts from other countries who participated in this study were generally of the opinion that the positive approach towards sexual expression taken in some European countries provides significant benefits for patients, particularly those who remain institutionalised for a long time, in terms of psychological well-being and therapeutic recovery. Possible explanations for the UK approach include the risk of litigation, fears in relation to capacity, risks of sexual assault or exploitation and onsite prostitution, and it has been suggested that such concerns may cause UK hospitals to err always on the side of caution and prohibition [2]. Another factor is anticipation of the negative media attention likely to accompany a more permissive policy. This is inevitably linked to a more general public disapproval of patients’ sexual expression [8] and reflects a wider prejudice against patients in forensic hospitals as being less worthy of having ‘privileges’ and requiring greater social control [23].

Negative assumptions such as these (which may themselves reflect public opinion) have led to calls for a determined campaign to promote a positive public discourse on patients’ sexual needs, rights and entitlements [16]. Indeed, Perlin and Lynch [6] have suggested that *“It is time for a radical change of perspective and attitude in how society views sexuality, and right to express that sexuality…”.* In our sample, however, Belgium and Switzerland were the only countries providing staff with specific training on patient sexual expression. Such changes in policy would need to be accompanied by a comprehensive training of clinical staff to help them recognise and respond more thoughtfully to patients’ sexual and intimacy needs, taking into account any differences in sexual and emotional needs between male and female patients. Staff may also need help in distinguishing between consensual and coercive sexual relationships between those in their care. Benefits of such policy change might be improvement in their relationships and authority with patients.

### Limitations

The high level of training and extensive experience of the expert participants in this study adds weight to the confidence and authority of their responses. The study has certain limitations, however. First, some participants made their responses on the basis of a specific local policy which may not be representative of the whole country. Second, forensic healthcare in many European countries is delivered in both high secure and medium secure units in varying proportions; this will likely be reflected in the expert participants’ responses, making it more difficult to compare some of the findings between one country and another. Third, the countries targeted formed only a proportion of all the European member states and so the findings are neither comprehensive nor necessarily representative of the whole European Union; this is entirely in keeping with the study’s stated aim which was to focus on the diversity of the various approaches. It is important to note though that there was a bias towards ‘Western European’ countries in those included in this study and only one country did not form part of the European Economic Area (EEA). It would be worthwhile to explore specifically the challenges faced by countries outside the EEA, and more specifically those of former USSR countries but this was beyond the scope of this study.

Looking to the future, we conclude that more attention needs to be given to the prospect that most patients will eventually leave hospital and be rehabilitated back into the community, and that any repressive sexual policies experienced whilst in detention will have left a marked impact on their sexuality which they will subsequently carry out with them. It would seem helpful to encourage discussion of these and other related topics at conferences, workshops and forums across Europe to expand awareness horizons and pool knowledge.

Following on from this exploratory study, further research might usefully focus on a wider range of countries. Since the studies published to date have tended to focus on the clinician’s point of view and be largely concerned with formal hospital policy, it will be important to examine the views of the patients themselves on issues such as how significant sexual expression is for them, and how much the hospital reflects their needs in its protocols. Such articulation would be crucial when reforming policy, would give patients more autonomy and would empower them in voicing their needs in what is effectively their home for several years.

## Additional files

10.1186/s13033-016-0037-y Questionnaire, list of questions used in the online survey.
10.1186/s13033-016-0037-y Interview Questions, list of questions used in the telephone interviews.

## References

[1] Brown SD, Reavey P, Kanyeredzi A, Batty R (2014). Transformations of self and sexuality: psychologically modified experiences in the context of forensic mental health. Health.

[2] Gordon H, Lindqvist P (2007). Forensic psychiatry in Europe. Psychiatr Bull.

[3] Völlm B, Edworthy R, Furtado V (2014). EPA-0119—Characteristics and needs of long stay patients in high and medium secure forensic psychiatric care: implications for service organisation. Eur Psychiatry.

[4] McCann E (2003). The expression of sexuality in people with psychosis: breaking the taboos. J Adv Nurs.

[5] Bartlett P, Mantovani N, Cratsley K, Dillon C, Eastman N (2010). ‘You may kiss the bride, but you may not open your mouth when you do’: policies concerning sex, marriage and relationships in English forensic psychiatric facilities. Liverpool Law Rev.

[6] Perlin ML, Lynch AJ (2014). ‘All his sexual patients’: persons with mental disabilities and the competence to have sex. Wash Law Rev.

[7] Howard League for Penal Reform. Consensual sex among men in prison. Briefing paper 1. London: Howard League for Penal Reform; 2013.

[8] Salize HJ, Dressing H (2007). Placement and treatment of mentally ill offenders–basic concepts and service provision in European Union Member States [Article in German]. Psychiatr Prax.

[9] Taylor PJ, Graf M, Schanda H, Völlm B (2012). The treating psychiatrist as expert in the courts: is it necessary or possible to separate the roles of physician and expert?. Crim Behav Ment Health.

[10] Marle HV (2000). Forensic psychiatric services in the Netherlands. Int J Law Psychiatry.

[11] Müller-Isberner R, Freese R, Jöckel D, Cabeza SG (2000). Forensic psychiatric assessment and treatment in Germany. Int J Law Psychiatry.

[12] Völlm B (2007). Compulsory psychiatric detention and treatment in Finland. Psychiatr Bull.

[13] Dein K, Williams PS (2008). Relationships between residents in secure psychiatric units: are safety and sensitivity really incompatible?. Psychiatr Bull.

[14] Wright ER, McCabe H, Kooreman HE. Institutional capacity to respond to the ethical challenges of patient sexual expression in state psychiatric hospitals in the United States. J Ethics Ment Health. 2012; 7:1–5. http://www.jemh.ca/issues/v7/. Accessed 10 May 2015.

[15] Lew K (2014). Study on International Attitudes to Sexual Expression in Forensic Settings (Masters dissertation).

[16] McCann E (2003). Exploring sexual and relationship possibilities for people with psychosis: a review of the literature. J Psychiatr Ment Health Nurs.

[17] Mossman D, Perlin ML, Dorfman DA. Sex on the wards: conundra for clinicians. J Am Acad Psychiatry Law. 1997; 25(4):441-60. http://www.jaapl.org. Accessed 10 May 2015. 9460033

[18] Hales H, Romilly C, Davison S, Taylor PJ (2006). Sexual attitudes, experience and relationships amongst patients in a high security hospital. Crim Behav Ment Health.

[19] Bruan V, Clarke V (2006). Using thematic analysis in psychology. Qual Res Psychol.

[20] Marques JF, McCall C. The application of interrater reliability as a solidification instrument in a phenomenological study. Qualitative Rep. 2005; 10(3):439–62. http://www.nova.edu/ssss/QR/QR10-3/marques.pdf. Accessed 10 May 2015.

[21] Tapp J, Warren F, Fife-Shaw C, Perkins D, Moore E (2013). What do the experts by experience tell us about ‘what works’ in high secure forensic inpatient hospital services?. J Forens Psychiatry Psychol.

[22] Ruane J, Hayter M (2008). Nurses’ attitudes towards sexual relationships between patients in high security psychiatric hospitals in England: an exploratory qualitative study. Int J Nurs Stud.

[23] Perlin ML. ‘Everybody is making love/or else expecting rain’: considering the sexual autonomy rights of persons institutionalized because of mental disability in forensic hospitals and in Asia. Wash Law Rev. 2008;83: 481–512. http://www.ssrn.com/abstract=1337164. Accessed 10 May 2015.

