# Supplementary material for: Policies on sexual expression in forensic psychiatric settings in different European countries
Source: Int J Ment Health Syst. 2016 Feb 3;10:5. doi: 10.1186/s13033-016-0037-y (PMC4741020; doi:10.1186/s13033-016-0037-y)
Supplement: Supplementary file 1 — 10.1186/s13033-016-0037-y Questionnaire, list of questions used in the online survey. [file 13033_2016_37_MOESM1_ESM.docx]

**Questionnaire**

This is an invitation to take part in a research study on national policies/guidance on sexual expression in secure forensic-psychiatric settings in different European countries.

If you decide to participate, you will be required to complete an online survey describing your country’s national policy (or agreed practice or local policies within specific institutions if a national policy does not exist) on sexual expression in secure forensic settings, including how you deal with particular situations such as relationships between patients. If further specific information is required a follow up interview by phone/Skype may be requested but there is no requirement that you take part in this.

Participation in this study is totally voluntary and you are under no obligation to take part. You are free to withdraw at any point before or during the study. Responses are anonymous and all data collected will be kept confidential and used for research purposes only. It will be stored in compliance with the UK Data Protection Act.

The results will form part of my MSc Psychology dissertation at the University of Nottingham. My supervisors are Dr Birgit Völlm, Clinical Associate Professor in Forensic Psychiatry and Dr Stephanie McDonald, Teaching Associate.

If you would like to participate, please complete the following questions (you may skip questions if you don’t wish to answer them but please try to answer as many questions as you can). There is a mixture of open and closed questions.

**Consent**

Please answer these questions:

• Have you read and understood the Information Sheet? YES/NO

• Have you had the opportunity to ask questions about the study? YES/NO

• Have all your questions been answered satisfactorily? YES/NO

• Do you understand that you are free to withdraw from the study? YES/NO

(at any time and without giving a reason)

• Do you agree to take part in the study? YES/NO

By clicking the button above I indicate that the study has been explained to me to my satisfaction, and I agree to take part. I understand that I am free to withdraw at any time.

For the purposes of this survey, the term ‘sexual expression’ refers to any form of intimacy including sexual intercourse (heterosexual and homosexual) within a forensic-psychiatric setting.

**Section A: General Information**

1. Which country do you work in?

_____________________________________________________________________________

1. Is there any national policy/guidance on sexual expression in forensic-psychiatric settings in your country?

Policy [ ] Guidance [ ] Neither [ ]

1. If not, is there nevertheless shared practise with regards to sexual expression in forensic-psychiatric settings?^^[[1]](#footnote-1)^^

Yes [ ] No [ ]

**Please answer the following questions only if there is NO national policy, guidance or shared practise.**

1. Is there a local (e.g. hospital specific) policy on sexual expression in a forensic psychiatric setting that you are familiar with?

Yes [ ] No [ ]

1. Which local area or hospital does this policy cover?

_______________________________________________________________________________

If the answer to above questions 2., 3. and 4. is ‘no’ the questionnaire is to be terminated here. Thank you for your participation.

**Please answer the following questions in relation to either a national policy, guidance or shared practice or the local policy referred to in the last question.**

**Section B: Applicability of Policy^^[[2]](#footnote-2)^^**

1. Is the policy the same for all forensic unit types, e.g. units of different level of security?

Yes [ ] No [ ] Don’t know [ ]

Further comments, e.g. if it does not apply equally...

If the policy differs according to unit type, please answer the following questions in relation to high secure units only.

1. Does the policy apply equally to heterosexual and homosexual relationships?

Yes [ ] No [ ] Don’t know [ ]

Further comments, e.g. if it does not apply equally...

1. Do the rules on sexual expression and relationships apply to all patients the same or do they differ depending on e.g. diagnosis, offending history or other patient characteristics?

Same [ ] Differ [ ] Don’t know [ ]

Further comments, e.g. if they differ…

**Section C: Sexual intercourse**

1. What is the overall direction of the policy?

Sexual intercourse is prohibited [ ] Sexual relationships are to be actively discouraged [ ]

Sexual relationships are permitted in certain circumstances [ ] Other [ ]

Please specify________________

Further comments…

1. If sexual relationships are prohibited, what are the main reasons for this?

_____________________________________________________________________________________________________________________________________________________________________________________________________________________________________________

1. If sexual relationships are not explicitly prohibited – what type of relationships are allowed?

Between patients [ ] Between patient and previously existing long-term partner [ ]

Between patient and new partner outside the institution [ ]

Further comments…

1. If sexual relationships are not explicitly prohibited, in practise – how many patients do you think regularly (by this we mean more than twice a year) engage in sexual relationships with:

Other patients Less than 1% [ ] Between 1 and 10% [ ] Between 10 and 30% of patients [ ] More than 30% of patients [ ] Don’t know [ ]

With their long-term partner Less than 1% [ ] Between 1 and 10% [ ] Between 10 and 30% of patients [ ] More than 30% of patients [ ] Don’t know [ ]

With a new partner outside the institution Less than 1% [ ] Between 1 and 10% [ ] Between 10 and 30% of patients [ ] More than 30% of patients [ ] Don’t know [ ]

Further comments…

1. According to this policy are patients able to see prostitutes?

Yes [ ] No [ ] Don’t know [ ]

Further comments…

**Section D: Other forms of sexual expression**

1. Does this policy make specific reference to sexually inappropriate behaviour?

Yes [ ] No [ ] Don’t know [ ]

Further comments, e.g. if it does make specific reference…

1. Does the policy allow the following types of sexual expression/affection?

Kissing Yes [ ] No [ ] Depending on circumstances [ ]

Don’t know [ ]

Hugging Yes [ ] No [ ] Depending on circumstances [ ]

Don’t know [ ]

Caressing/stroking Yes [ ] No [ ] Depending on circumstances [ ]

Don’t know [ ]

Massage/back rub Yes [ ] No [ ] Depending on circumstances [ ]

Don’t know [ ]

Holding hands Yes [ ] No [ ] Depending on circumstances [ ]

Don’t know [ ]

Masturbation Yes [ ] No [ ] Depending on circumstances [ ]

Don’t know [ ]

Further comments…

1. Does the policy allow patients to access the following?

Sexually explicit videos/DVDs Yes [ ] No [ ] Depending on circumstances [ ]

Don’t know [ ]

Adult magazines Yes [ ] No [ ] Depending on circumstances [ ]

Don’t know [ ]

Erotic novels Yes [ ] No [ ] Depending on circumstances [ ]

Don’t know [ ]

Pornographic websites Yes [ ] No [ ] Depending on circumstances [ ]

Don’t know [ ]

Further comments…

1. Does the policy allow non-sexual relationships between patients?

Yes [ ] No [ ] Depending on circumstances [ ] Don’t know [ ]

Further comments…

1. Does the policy give patients the right to marry?

Yes [ ] No [ ] Depending on circumstances [ ] Don’t know [ ]

Further comments…

1. Does the policy consider pregnancy?

Yes [ ] No [ ] Depending on circumstances [ ] Don’t know [ ]

Further comments…

**Section E: Patient/Visitor Interaction**

1. Are all wards segregated by gender?

Yes [ ] No [ ] Don’t know [ ]

1. If so, do male and female patients ever interact with each other within the unit?

Yes [ ] No [ ] Don’t know [ ]

Further comments, e.g. if they do interact with each other…

1. Does this policy allow peers unsupervised access to each other’s bedrooms?

Yes [ ] No [ ] Depending on circumstances [ ] Don’t know [ ]

Further comments…

1. Does this policy allow peers supervised access to each other’s bedrooms?

Yes [ ] No [ ] Depending on circumstances [ ] Don’t know [ ]

Further comments…

1. Does this policy allow visitors unsupervised access to patients’ bedrooms?

Yes [ ] No [ ] Depending on circumstances [ ] Don’t know [ ]

Further comments…

1. Does this policy allow visitors supervised access to patients’ bedrooms?

Yes [ ] No [ ] Depending on circumstances [ ] Don’t know [ ]

Further comments…

1. Are patients able to use conjugal visiting suites? (i.e. a place where visitors can have unsupervised contact with the detained person)

Yes [ ] No [ ] Depending on circumstances [ ] Don’t know [ ]

Further comments…

1. Are visits by friends or relatives from the outside always supervised by staff?

Yes [ ] No [ ] Depending on circumstances [ ] Don’t know [ ]

Further comments…

**Section F: Resources Available**

1. Is relationship counselling available to patients?

Yes [ ] No [ ] Depending on circumstances [ ] Don’t know [ ]

Further comments…

1. Is sexual education available to patients?

Yes [ ] No [ ] Depending on circumstances [ ] Don’t know [ ]

Further comments…

1. Does this policy allow access to condoms/contraception to patients?

Yes [ ] No [ ] Depending on circumstances [ ] Don’t know [ ]

Further comments…

**Section G: Staff**

1. Does this policy comment on patient - staff relationships?

Yes [ ] No [ ]

Further comments, e.g. if the policy does comment…

1. Are all staff made aware of the policy on sexual expression?

Yes [ ] No [ ] Don’t know [ ]

Further comments…

1. Do staff have training on matters related to sexuality and sexual expression?

Yes [ ] No [ ] Don’t know [ ]

Further comments, e.g. if staff do get training…

**Section H: Patient Involvement**

1. Is the policy/guidance readily available for patients to see?

Yes [ ] No [ ] Don’t know [ ]

Further comments (insert free text box here)

1. Was the patient perspective considered in the drafting of the policy?

Yes [ ] No [ ] Don’t know [ ]

Further comments…

1. Additional comments

____________________________________________________________________________________________________________________________________________________________________________________________________________________________________________________________________________________________________________________________________

1. By this we mean that it could be expected that the practise would be the same or largely similar in different units, e.g. with regards to what type of relationships, sexual expression, etc. are allowed [↑](#footnote-ref-1)
2. We are using ‘policy’ here to keep the text brief but we mean policy, guidance or shared practise [↑](#footnote-ref-2)
