# Supplementary material for: Policies on sexual expression in forensic psychiatric settings in different European countries
Source: Int J Ment Health Syst. 2016 Feb 3;10:5. doi: 10.1186/s13033-016-0037-y (PMC4741020; doi:10.1186/s13033-016-0037-y)
Supplement: Supplementary file 2 — 10.1186/s13033-016-0037-y Interview Questions, list of questions used in the telephone interviews. [file 13033_2016_37_MOESM2_ESM.docx]

**Interview Questions**

Germany Interview Questions:

1. May I start by asking you to briefly describe the institution you currently work in and your role within this institution?
2. Approximately how many male and how many female patients are there?
3. To clarify, is there shared practice or a policy regarding sexual expression in Haina, Germany?
4. Could you explain the practice/policy briefly to me please?
5. You say in the survey sexual relationships are permitted in certain circumstances. When would sexual relationships not be permitted? The reason for this?
6. When relationships are not allowed – do such relationships nevertheless happen, and how is this managed? What might be the consequences for patients if they engage in such relationships?
7. You say that ‘depending on circumstances’ kissing, hugging, etc. would be allowed. Does this relate to patients among themselves or patients and outside partners?
8. You say sexual relationships between patient-patient and patients and outside partners are allowed. How is this managed?
9. Are conjugal visiting suites available to patients?
10. Are patients and their partners allowed private time in their bedroom or some other designated room?
11. How does this work in practice?
12. Have any problems, and, if so, which kind of problems, ever arisen from this policy?
13. You say you allow access to condoms/contraception – what is the procedure for this? When would they use them?
14. What is the general response from patients regarding the rule on sexual expression? Have patients ever challenged or expressed concerns about the practise?
15. What is the general response from staff regarding the practicality of the policy?
16. Are there any other areas of the practice you described and how this works in practice that you would like to highlight?
17. Do you think there is a stigma attached to the subject of sexuality and relationships?
18. Do you think there are conflicting ideas re sexual expression and that is why there is no national policy?
19. What do you think could be done to improve issues around sexuality, relationships and intimacy in your country?

Netherlands Interview Questions

1. May I start by asking you to briefly describe the institution you currently work in and your role within this institution?
2. I understand there is a shared practice amongst forensic-psychiatric hospitals with regards to sexual expression in patients in the Netherlands. Could you explain the practise briefly to me, please?
3. To clarify, are there also local policies that are hospital specific?
4. You mentioned in the questionnaire that higher secure units are more strict than medium secure units – could you give more detail about this please?
5. You say patients can have sexual intercourse during visits based on screening of the partners – what is the process for this?
6. You say relationships between patients are not allowed. What are the reasons behind this?
7. If sexual contact nevertheless did happen on the unit between patients, how is such a situation managed? Has this ever happened? What might be the consequences if they engage in such relationships?
8. Mentioned in the survey is that patients in some cases can marry – In what cases would they not be allowed to marry? What is the reason for this? Also, would they then be allowed to live together in the institution and, e.g., have intercourse together? Is this not contradictory to 6?
9. Do you think the practice is any different because the patients are male? Why?
10. You say you allow access to condoms/contraception – what is the procedure for this? When would they use them?
11. I heard that patients in the Netherlands are allowed to have access to prostitutes. Is this right? Or was this the case previously?
12. What is the general response from patients regarding the rule on sexual expression? Have patients ever challenged or expressed concerns about the practice?
13. What is the general response from staff regarding the practicality of the policy?
14. Are there any other areas of the practice you described and how this works in practice that you would like to highlight?
15. Do you think there is a stigma attached to the subject of sexuality and relationships?
16. Do you think there are conflicting ideas re sexual expression and that is why there is no national policy?
17. What do you think could be done to improve issues around sexuality, relationships and intimacy in your country?

Switzerland Interview Questions

1. May I start by asking you to briefly describe the institution you currently work in and your role within this institution?
2. Approximately how many male and how many female patients are there?
3. I understand there is shared practice with regards to sexual expression in patients in Switzerland.

Could you explain this practice briefly to me, please?

With regards to sexual relationships (heterosexual or homosexual), are they allowed between patients in forensic-psychiatric institutions? …between patients and partners outside the institution (e.g. husband and wife)?

1. You say relationships between patients are actively discouraged. What are the reasons behind this?
2. Do such relationships nevertheless happen, and how is this situation managed? What might be the consequences for patients if they engage in such relationships?
3. You say that ‘depending on circumstances’ kissing, hugging, etc. would be allowed. Does this relate to patients among themselves or patients and outside partners?
4. You say sexual relationships between patients and outside partners are allowed. How is this managed? Are patients and their partners allowed private time in their bedroom or some other designated room? How does this work in practice? Have any problems, and, if so, which kind of problems, ever arisen from this policy?
5. In the questionnaire you state the shared practice considers pregnancy – however, if it discourages relationships – how is a patient likely to get pregnant? What is the procedure for this? How often does it happen?
6. Allows access to condoms/contraception – what is the procedure for this? Also, if relationships are discouraged and patients always supervised when would they use these?
7. Confirm if sexual education is available to patients (answered yes and no in the questionnaire).
8. What is the general response from patients regarding the rule on sexual expression?
9. Have staff ever expressed any concerns about the practice, and, if so, what were they?
10. Are there any other areas of the practice you described and how this works in practice that you would like to highlight?
11. Do you think there is a stigma attached to the subject of sexuality and relationships?
12. Do you think there are conflicting ideas re sexual expression and that is why there is no national policy?
13. What do you think could be done to improve issues around sexuality, relationships and intimacy in your country?

UK High Secure Interview Question

1. May I start by asking you to briefly describe the institution you currently work in and your role within this institution?
2. I understand there is guidance on patient relationships at Rampton that ensures the effective management of relationships while recognising that forming relationships is a normal human activity.
   1. Could you explain the guidance briefly to me please?
   2. So, with regards to sexual relationships (heterosexual or homosexual) between patients they are not allowed, is this right?
   3. Could you tell me the main reasons for this?
   4. How about relationships between patients and partners outside the institution (e.g. husband and wife)? Would patients be able to have unsupervised contact within the unit with their partners, which may include sexual activities?
   5. If sexual contact nevertheless happen on the unit, how is such a situation managed? Has this ever happened? What might be the consequences for patients if they engage in such relationships?
   6. In your experience how many patients would you estimate currently are in a sexually active relationship with a) another patient, b) a partner outside the institution? How about emotional, but non-sexual relationships?
   7. Would they be able to engage in sexual activities outside the hospital (i.e. whilst on leave)?
3. Is sexual education available to patients?
4. Is relationship counselling available to patients?
5. Were patients involved in the development of the policy? Have patients ever challenged or expressed concerns about the policy?
6. How about staff? Have they ever expressed any concerns about the policy? In what way?
7. Are there any other areas of the policy you described and how this works in practice that you would like to highlight?
8. Rampton also have a procedure for ‘access to sexually explicit magazines’. Could you explain this briefly and the reason behind it?
   1. Do the same rules apply to sexually explicit videos/DVDs, erotic novels and pornographic websites?
9. I believe Rampton also has a procedure for ‘safeguarding vulnerable adults’ to provide a guide for staff in managing all staff-patient relationships. Would you be able to tell me the main points of this policy on this please? Has there ever been a time where staff have not adhered to this?
10. In our international policy review of so far we have found that all other countries allow or even encourage maintaining relationships, including sexual, relationships between patients and outside partners, in particular if these relationships are long-standing. A number of other countries also have a permissive approach to patient-patient relationships and some even to access to prostitutes. The reasons for this approach are the human rights of the patients where it is seen as disproportionately restrictive to not allow patients any means of sexual expression, often for many years. Respondents from other countries have also noted that maintaining or developing relationships with partners, in particular outside the institution and a fulfilled sex life can be a very positive factor in a patient’s recovery. A number of other countries facilitate sexual contact through the use of conjugal suites and very few problems have been reported with the approach.
    1. Were you aware of these different practices and why do you think this area is handled so differently in the UK?
11. Do you think there is a stigma attached to the subject of sexuality and relationships?
12. Do you think there are conflicting ideas re sexual expression and that is why there is not a national policy?
13. What do you think could be done to improve issues around sexuality, relationships and intimacy in your country?

UK Medium Secure Interview Questions

1. May I start by asking you to briefly describe the institution you currently work in and your role within this institution?
2. I understand there is a policy on patient relationships (which you have sent me) at the John Howard Centre/East London NHS Foundation Trust that recognises the importance of relationships, including sexual ones, for the patients’ recovery.
   1. Could you explain the policy briefly to me, please?
   2. So, with regards to sexual relationships (heterosexual or homosexual) between patients, they are not allowed within the unit, is that right?
   3. Could you tell me the main reasons for this?
   4. How about relationships patients and partners outside the institution (e.g. husband and wife)? Would patients be able to have unsupervised contact within the unit with their partners, which may include sexual activities?
   5. If sexual contact nevertheless happen on the unit, how is such a situation managed? Has this ever happened? What might be the consequences for patients if they engage in such relationships?
   6. In your experience how many patients would you estimate currently are in a sexually active relationship with a) another patient, b) a partner outside the institution? How about emotional, but non-sexual relationships?
   7. Where would they be able to engage in sexual activities if they are not allowed on the unit?
3. Were patients involved in the development of the policy? Have patients ever challenged or expressed concerns about the policy?
4. How about staff? Have they ever expressed any concerns about the policy? In what way?
5. Are there any other areas of the policy you described and how this works in practice that you would like to highlight?
6. In our international policy review of so far we have found that all countries allow or even encourage maintaining relationships, including sexual, relationships between patients and outside partners, in particular if these relationships are long-standing. A number of other countries also have a permissive approach to patient-patient relationships and some even to access to prostitutes. A number of other countries facilitate sexual contact through the use of conjugal suites and very few problems have been reported with the approach.
   1. Were you aware of these different practices, in particular the use of conjugal suites, and why do you think, this area is handled differently in the UK?
7. Do you think there is a stigma attached to the subject of sexuality and relationships?
8. Do you think there is conflicting ideas re sexual expression and that is why there is not a national policy?
9. What do you think could be done to improve issues around with sexuality, relationships and intimacy in your country?
